# Characteristics of primary and immortalized fibroblast cells derived from the miniature and domestic pigs

**DOI:** 10.1186/1471-2121-8-20

**Published:** 2007-06-01

**Authors:** Ho-Yeon Oh, Xun Jin, Jong-Geun Kim, Myung-Joo Oh, Xumin Pian, Jun-Mo Kim, Moon-Seok Yoon, Chae-Ik Son, Young Sik Lee, Ki-Chang Hong, Hyunggee Kim, Yun-Jaie Choi, Kwang Youn Whang

**Affiliations:** 1 Division of Biotechnology, College of Life Sciences and Biotechnology, Korea University, Seoul, Korea; 2 Division of Animal Biotechnology, Seoul National University, Seoul, Korea; 3 National Veterinary and Quarantine Service, Incheon, Korea

## Abstract

**Background:**

The pig, *Sus scrofa domestica *includes both the miniature and commercial domestic breed. These animals have influenced the human life and economies and have been studied throughout history. Although the miniature breeds are more recent and have increasingly been used in a variety of biomedical studies, their cell lines have rarely been established. Therefore, we sought to establish primary and immortal cell lines derived from both the miniature and domestic pig to better enable insight into possible *in vivo *growth differences.

**Results:**

The *in vitro *lifespan of primary domestic pig fibroblast (PF) and miniature pig fibroblast (MPF) cells using a standard 3T3 protocol was determined. Both of the primary PF and MPF cells were shown to have a two-step replicative senescence barrier. Primary MPF cells exhibited a relatively shorter lifespan and slower proliferation rate compared to those of primary PF cells. Beyond senescence barriers, lifespan-extended PF and MPF cells were eventually established and indicated spontaneous cellular immortalization. In contrast to the immortalized PF cells, immortal MPF cells showed a transformed phenotype and possessed more frequent chromosomal abnormalities and loss of p53 regulatory function. The lifespan of primary MPF and PF cells was extended by inactivation of the p53 function using transduction by SV40LT without any detectable senescent phenotype.

**Conclusion:**

These results suggest that p53 signaling might be a major determinant for the replicative senescence in the MPF cells that have the shorter lifespan and slower growth rate compared to PF cells *in vitro*.

## Background

Research using *in vitro *cell culture methods has a number of limitations to a complete understanding of biological systems *in vivo*. The primary somatic cells, however, are valuable tools to enable the study of a variety of cellular and biochemical functions under tightly controlled experimental conditions. One limitation to primary somatic cell use that must be managed is their finite proliferative capacity due to permanent growth arrest known as replicative senescence [1]. Replicative senescence is known to be triggered by two inter-dependent mechanisms; one is activation of two tumor suppressor pathways (p16^INK4a^/RB and ARF/p53, [2]), the second is a shortening of the telomeres due to an end-replication problem during chromosome replication [3,4]. To overcome these limitations, much effort has been put into the establishment of immortalized cell lines that have an unlimited replicative potential and normal cellular functions [5-10]. The loss of a tumor suppressor pathway, such as inactivation of p53 and Rb by simian virus 40 large T antigen (SV40LT), bypasses senescent-mediated growth arrest and ultimately extends cellular lifespan [9,11,12]. The maintenance of telomere length by the overexpression of human telomerase (hTERT) is known to avoid replicative senescence and to establish immortalized cell lines from various species [13-17].

Because of the close physiological and anatomical similarities with humans compared to other non-rodent species, miniature pig breeds have increasingly been used as models for research in physiology, immunology, toxicology, nutrition, drug metabolism, and various diseases. Although the miniature pig breeds are currently used as general surgical models for many organs, for cardiovascular research, digestive system models, transplantation and xenografts [18-21], their primary and immortal cell lines have rarely been established. Therefore, we have conducted research to establish primary and immortal cell lines derived from miniature (MPF cells) and domestic pigs (PF cells) to use as *in vitro *model systems to explore and better understand the cellular and biochemical mechanisms that underlie *in vivo *physiological events.

## Results

### *In vitro *growth characteristics of primary fibroblast cells derived from the miniature and domestic pig

**Figure 1:**
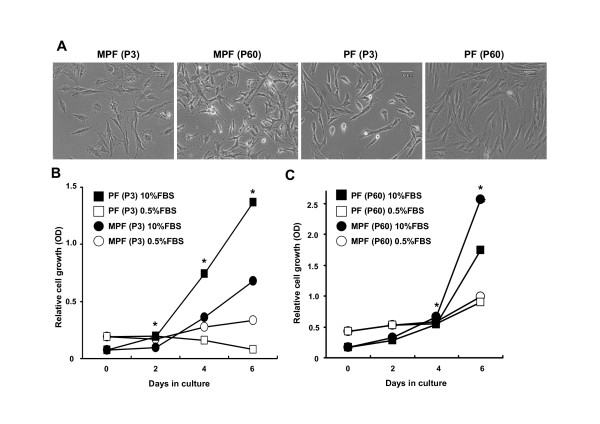
**Cell morphology and cell growth rates of MPF and PF cells**. **A**. Representative cell morphology of MPF and PF cells in early (passage 3; P3) and late passages (passage 60; P60) (40× magnifications). **B**. Relative growth rates of MPF and PF cells at an early passage (P3) grown in 10% and 0.5% FBS-DMEM as determined by staining cells with crystal violet solution every 2 days for 6 days. Data shown are means ± SEM (n = 3). Asterisk (*) indicates a significant difference between MPF and PF cells grown in the 10% FBS-DMEM (p < 0.05). **C**. Relative growth rates of MPF and PF cells at a late passage (P60) grown in 10% and 0.5% FBS-DMEM. Data shown are means ± SEM (n = 3). Asterisk (*) indicates a significant difference between MPF and PF cells grown in the 10% FBS-DMEM (p < 0.05).

**Figure 2:**
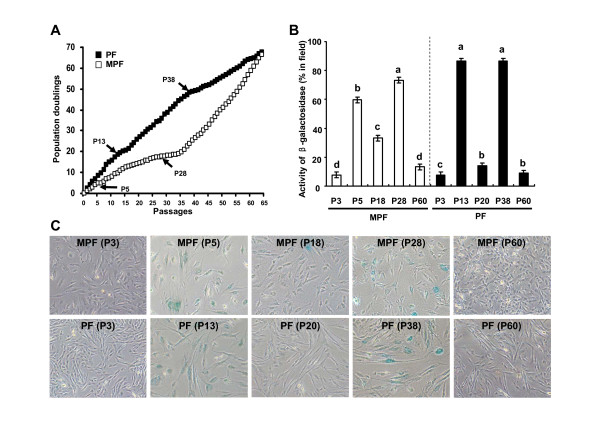
***In vitro *lifespan and senescence-associated β-galactosidase activity of MPF and PF cells**. **A**. Cumulated cell population doubling rates were determined in primary MPF and PF cells using a standard 3T3 cell culture protocol. **B**. Senescence-associated β-galactosidase activity (SA-β-gal)-positive cell numbers of the different passages MPF and PF cells. Data shown are means ± SEM (n = 3). The a, b, c, and d indicate significant differences (p < 0.05). **C**. The representative photographs (40× magnification) of different passages MPF and PF cells showing SA-β-gal activity.

We established two independent lines of primary and immortalized porcine fibroblast cells from miniature (MPF cells) and domestic (PF cells) pigs, respectively (Figure 1A). In the presence of 10% FBS, the growth rate of the early passage PF cells (P3) was shown to be more rapid than that of the MPF cells (P3), whereas both cells failed to proliferate in the presence of 0.5% FBS, thereby indicating that both cells require FBS for proliferation (Figure 1B). In contrast to primary cells, immortalized MPF cells (P60) grew faster than immortalized PF cells (P60) (Figure 1C), implying possible genetic alterations that enable to change in cellular growth property during immortalization process. We next determined the *in vitro *lifespan of primary MPF and PF cells using a standard 3T3 protocol. As shown in Figure 2A, there was a marked difference in the growth curves of the MPF and PF cells. For MPF cells, replicative senescence appeared at two distinct passages (P5 and P28) as judged by a flat morphology and senescence-associated β-galactosidase activity (SA-β-gal) (Figure 2B and 2C), and a growth rate was shown to decrease until passage 34, but rapidly increase after passage 35. It is noteworthy that less than 35% of MPF cells at passage 7 through passage 24 displayed SA-β-gal-positive (Figure 2B and 2C, data not shown). For the PF cells, the replicative senescence appeared at two distinct passages (P13 and P38). Taken together, the MPF cells grew at a slower rate and had a shorter *in vitro *lifespan compared to the PF cells. Since the MPF and PF cells, after passages 35 and 40, respectively, grew continuously without a detectable senescent phenotype, we speculate that both the MPF and PF cells by passage 60 would be spontaneously immortalized.

### p53 and p21^WAF1 ^expression and doxorubicin-resistant cell viability of primary and immortalized MPF and PF cells

**Figure 3:**
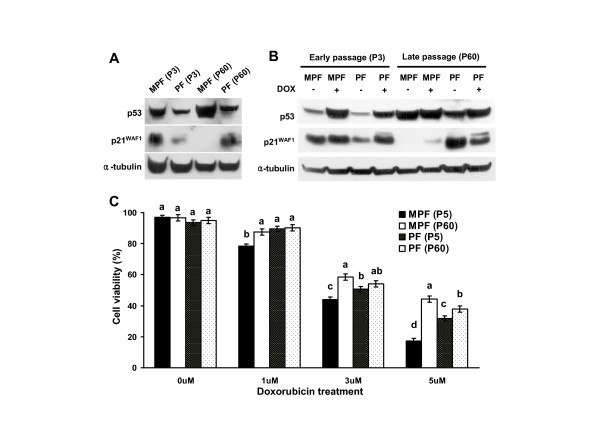
**p53 and p21^WAF1 ^expression and doxorubicin-resistant cell viability of primary and immortalized MPF and PF cells**. **A**. Expression of p53 and p21^WAF1 ^proteins in the primary and immortal MPF and PF cells. α-tubulin was used as a loading control. **B**. Expression of p53 and p21^WAF1 ^proteins in the primary and immortal MPF and PF cells grown in the absence or presence of doxorubicin (3 uM). α-tubulin was used as a loading control. **C**. Cell viability (%) of the primary and immortal MPF and PF cells grown in the absence or presence of doxorubicin (1, 3, and 5 uM) for 24 hr. The a, b, c, and d indicate significant differences (p < 0.05).

Since it has been well documented that p53 gene, a cell cycle checkpoint and tumor suppressor, was commonly inactivated in numerous immortal and transformed cells [22], we determined expression levels of p53 and p21^WAF1^, one of p53-downstream target genes, as well as biological function of p53 in the primary and immortalized MPF and PF cells. As shown in Figure 3A, expression level of p53 protein was found to be similar in the primary and immortalized PF cells, while p21^WAF1 ^was relatively upregulated in the immortalized PF cells compared to the primary PF cells. However, we think that expression levels of p53 and p21^WAF1 ^proteins might be more elevated in the immortalized PF cells compared to primary PF cells as normalized to α-tubulin level (P3 and P60 in Figure 3A). When primary PF cells were treated with doxorubicin (a DNA-damaging agent) that enables to stabilize and activate p53 protein, p53 protein level was shown to be markedly elevated with concomitant increase of p21^WAF1 ^(Figure 3B). Interestingly, the expression of p21WAF was relatively decreased in the immortalized PF cells treated with doxorubicin (Figure 3B).

However, like other immortalized or transformed cells, the expression of p53 protein was shown to be markedly elevated in the immortalized MPF cells, whereas p21^WAF1 ^protein was dramatically downregulated in these cells as compared to the primary MPF cells (Figure 3A). In addition, when primary MPF cells were treated with doxorubicin, p53 protein was shown to be markedly elevated in these cells with concomitantly slight increase of p21^WAF1^, whereas expression of the p53 protein was not changed by a DNA damage response, and p21^WAF1 ^protein was barely detectable in the immortal MPF cells, regardless of doxorubicin (Figure 3B). Furthermore, in the presence of doxorubicin (3 and 5 uM), the immortalized MPF cells were more resistant to cell death as compared to primary MPF cells (p < 0.05, Figure 3C), whereas immortalized PF cells showed slightly increased resistance to doxorubicin (only at 5 uM) as compared to primary PF cells (Figure 3C). Taken together, these results indicate that immortalized MPF cells, but not immortalized PF cells, may be defective for the p53 regulatory function.

### Transformed phenotype and increased chromosome abnormality in the immortalized MPF cell line

**Figure 4:**
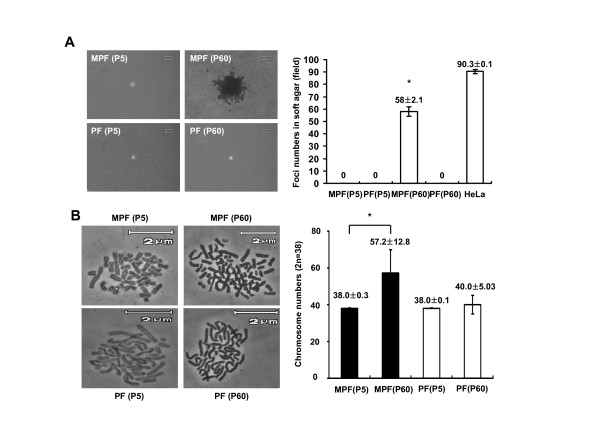
**Anchorage-independent growth and chromosome abnormalities of the immortal MPF cells**. **A**. Representative photographs showing primary and immortal MPF and PF cells grown under soft-agar culture conditions for 3 weeks (Left), and the foci numbers of primary and immortal MPF and PF cells grown in the soft-agar (Right). HeLa cells served as the positive control. The number shown in the graph are means ± SEM (n = 3). Asterisk (*) indicates a significant difference (p < 0.001). **B**. Representative photographs showing metaphase chromosomes of primary and immortal MPF and PF cells (400× magnifications; Left), and average number of chromosomes of primary and immortal MPF and PF cells (Right). The number shown in the graph are means ± SEM (n = 100). Asterisk (*) indicates a significant difference (p < 0.05).

To address whether primary and immortal MPF and PF cells have a transformed property, we studied the anchorage-independent growth of primary MPF and PF cells by growing the cells in a soft-agar culture condition. As shown in Figure 4A, immortal MPF (passage 60), but not immortal PF cells, were enabled to grow in the soft-agar, suggesting that the immortalized MPF cells should possess a transformed property. As determined chromosome abnormality of the primary and immortal MPF and PF cells by a karyotyping (Figure 4B), 81 of the 100 immortalized MPF cells have abnormal chromosome numbers of 2N = 57.2 on average, while all of primary MPF and PF as well as immortalized PF cells were shown to have normal chromosome number of 2N = 38 on average (data not shown).

### Growth properties and *in vitro *lifespan of cells transduced with SV40LT and hTERT

**Figure 5:**
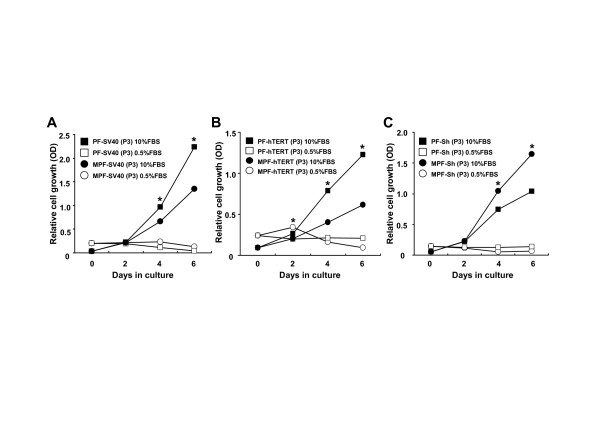
**Growth rates of primary MPF and PF cells transduced with SV40LT, hTERT and SV40LT+hTERT**. Relative growth rates of the MPF and PF cells transduced with SV40LT (**A**), hTERT (**B**) and SV40LT+hTERT (**C**) grown in DMEM supplemented with either 10% FBS or 0.5% FBS. Data shown are means ± SEM (n = 3). Asterisk (*) indicates a significant difference in the cells grown in the 10% FBS-DMEM (p < 0.05).

**Figure 6:**
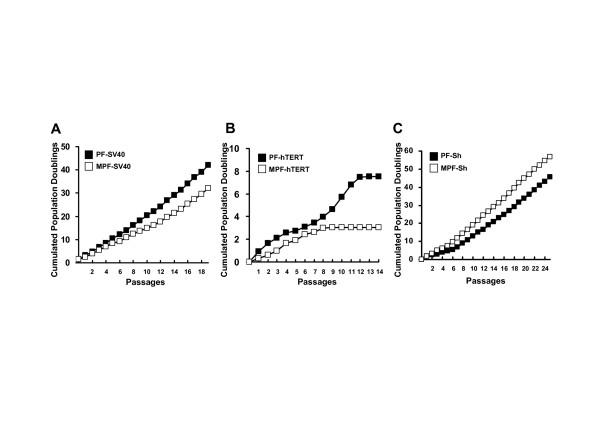
***In vitro *lifespan of MPF and PF cells transduced with SV40LT, hTERT and SV40LT+hTERT**. Cumulated population doubling rates of the MPF and PF cells transduced with SV40LT (**A**), hTERT (**B**) and SV40LT+hTERT (**C**) are determined by a standard 3T3 cell culture protocol. Data from three experiments are shown as means (n = 3).

Cellular immortalization and transformation have been shown to associate with loss of p53 and gain of telomerase activity [23,24]. Therefore, we transduced SV40LT (one of the p53 inactivators, [25]) and hTERT (human telomerase catalytic subunit) into primary MPF and PF cells in order to determine a possible causal role of the activated p53 or telomerase activity for a shorter lifespan and earlier senescent-mediated growth arrest of the primary MPF cells as compared to primary PF cells. Growth rate of the SV40LT- or hTERT-transduced PF cells was shown to be faster than that of the SV40LT- or hTERT-transduced MPF cells in the presence of 10% FBS, whereas these cells failed to proliferate in the presence of 0.5% FBS (Figure 5A and 5B). In contrast, the SV40LT+hTERT-transduced MPF cells were shown to proliferate relatively faster than SV40LT+hTERT-transduced PF cells in the presence of 10% FBS, whereas these cells failed to grow in the presence of 0.5% FBS (Figure 5C). In examining their *in vitro *lifespan by the 3T3 protocol, both SV40LT-transduced MPF and PF cells proliferated continuously and without any detectable senescent phenotype (Figure 6A), whereas transduction of hTERT into primary MPF and PF cells failed to extend their *in vitro *lifespan (Figure 6B). Interestingly, the hTERT-transduced MPF and PF cells were shown to become senescent at passages 9 and 13, and to enter crisis stage from passages 10 and 14, respectively. Meanwhile, transduction of SV40LT+hTERT into primary MPF and PF cells, like transduction of SV40LT alone, exhibited an extension of their *in vitro *lifespan (Figure 6C). Of interest, the growth rate of the SV40LT+hTERT-transduced MPF cells were shown to be faster than their counterpart PF cells. PF and MPF cells transduced with SV40LT or SV40LT+hTERT have continuously proliferated beyond passage 60 without any detectable senescent barriers and we are of the opinion that these cells should be immortalized.

Since hTERT, distinct from SV40LT, failed to extend cellular lifespan, we examined expression and relative activity of hTERT in the control and hTERT-transduced cells. Although hTERT mRNA was found to be expressed as determined by RT-PCR, the hTERT activity did not increase in the hTERT-transduced cells as compared to nontransduced cells (data not shown). Furthermore, we found that endogenous telomerase activity in the primary and immortal MPF and PF cells was similar to that in the primary human BJ fibroblast cells (data not shown). These results suggest that activation of the telomerase might not be necessary for cellular immortalization at least in the MPF cells of this study, whereas loss of p53 should be sufficient for immortalization and p53 activation in these cells might be a major determinant for the replicative senescence.

## Discussion

Although numerous immortalized cell lines have been established from various domestic animals, such as bovine, equine, ovine, avian, canine and porcine [10,15,26-29], here, we first report establishment of the spontaneous immortalized cells derived from miniature pigs.

One of the major regulatory pathways for replicative senescence is known to come from activation of p53, one of the best characterized cell cycle checkpoints and tumor suppressors [9,30]. p53 functions as transcriptional activator or repressor and plays a crucial role in cell proliferation and transformation by tightly regulating expression of the various cell cycle negative regulators and apoptosis-inducing factors such as p21^WAF1 ^and BAX, respectively [31-33]. It has been documented that p53 activity is markedly elevated in the senescent cells, whereas loss of p53 is sufficient to escape senescent barriers and ultimately become immortalized in a variety of cells [34-36].

A shortening of telomere length by inactivation of telomerase should also be associated with senescent-mediated growth arrest in a variety of species [9,36]. In normal somatic cells, the telomere shortening occurs in each cell division as a result of the end-replication problem of DNA polymerase [37]. Critically shortened telomeres trigger loss of chromosomal integrity and concomitantly activate DNA damage response that eventually results in irreversible cell cycle arrest (senescence) through activation of p53 function [38].

In the present study, the primary MPF cells showed slower growth rate and shorter *in vitro *lifespan compared to the primary PF cells. The shorter lifespan in the primary MPF cells might be caused by activation of p53 as judged by relative expression levels of p53 and p21^WAF1 ^(one of the p53-downsteam target genes). Although expression of p53 was elevated in immortalized MPF cells compared to their counterpart cells, expression of p21^WAF1 ^was shown to be dramatically decreased in these cells. However, there is a question of how p21^WAF1 ^is downregulated in the immortalized MPF cells possessing a significantly higher steady-state level of p53 protein. This converse expression pattern of p53 and p21^WAF1 ^in the immortalized MPF cells has been commonly observed in other immortalized cells [39,40]. It is also well-characterized that when p53 protein is inactivated by point mutation, its stability is known to increase owing to evasion of MDM2-dependent ubiquitin-mediated proteolysis [41]. Therefore, we speculate that p53 function in the immortalized MPF cells might be inactivated by point mutation.

In accordance with previous data [42], our results have indicated that the activation of p53 signaling should be a major determinant for the replicative senescence in the cells derived from pigs, because inactivation of the p53 signaling by SV40LT was shown sufficient for both primary MPF and PF cells to escape from the senescent barrier. However, we can not rule out that a variety of other genetic alterations, such as inactivation of Rb by SV40LT or less well characterized associations of p300/CBP and Bub1 with SV40LT, might be also involved in cellular immortalization [43]. Furthermore, since the spontaneously immortalized PF cells are recognized to possess functional p53 activity, it is also possible that cellular immortalization of pig cells might be occurred by external microenvironmental changes [44,45] or other endogenous genetic alterations such as loss of Ink4a/Arf tumor suppressor, without loss of p53 function [46].

In the case of telomerase activity, endogenous telomerase activity in the all primary and immortalized pig cells used in the present study was found to possess minimum basal levels that are similar to that in the human BJ fibroblast cells. Moreover, we failed to reconstitute telomerase activity in the PF and MPF cells by overexpression of human telomerase catalytic subunit (hTERT), although expression of exogenous hTERT in those cells was verified by RT-PCR (data not shown). A couple of research groups have demonstrated that most somatic cells derived from pigs, unlike human somatic cells, are known to possess endogenous telomerase activity [47,48]. However, recent reports have also demonstrated that endogenous telomerase activity was shown to be restricted in the particular tissues/organs of pigs, and introduction of hTERT failed to reconstitute telomerase activity in the cells derived from pig [30,49,50]. These results suggest that telomerase activity might be required for cellular immortalization in the pig cells, depending on cell types, tissue origins or culture conditions.

Since it has been documented that p53 protein is stabilized and activated through its phosphorylation under the various cellular insults such as DNA damage and oncogenic stresses [51], the relatively increased p53 level in the primary MPF cells of early passage (P3) compared to primary counterpart PF cells (P3) reflects that the primary MPF cells might be prone to possess relatively higher cellular stresses, compared to the primary PF cells. Among various cellular stresses that enable to activate p53, chromosome-spindle attachment might be improperly regulated in the primary MPF cells, by which a relatively higher steady-state level of the p53 protein would be maintained in these cells. This speculation is further supported in that cells containing abnormal chromosome numbers (3N) are dramatically increased in the immortalized MPF cells possessing inactivated p53 function, and deregulation of chromosome-spindle attachment could activate not only p53-dependent checkpoint pathway but also stimulate replicative senescence [52,53]. Therefore, we assume that the loss of the p53 activity in the immortalized MPF cells should increase chromosome abnormality, ultimately leading to cellular transformation.

## Conclusion

Collectively, the results of this study demonstrate a number of molecular and cellular biological differences between primary and immortalized cells derived from domestic and miniature pigs. Primary MPF cells showed relatively slower growth and shorter *in vitro *lifespan compared to the PF cells. Furthermore, activation of p53 function might be a major cause to display relatively earlier senescent phenotype and slower growth property in the MPF cells as compared to PF cells. In contrast to immortalized PF cells, immortalized MPF cells showed the loss of p53 function and the increased chromosomal abnormality, which might lead these cells to be transformed.

## Methods

### Animals

Göttingen miniature pigs were used as the miniature pig strain, while the three-way crossbred pig (Duroc, Landrace and Yorkshire) that is commercially used for pork production was our domestic strain. The Göttingen strain miniature breed was developed at Göttingen University in the 1960s by the cross-breeding of the Minnesota miniature pig initially with the Vietnamese pot-bellied pig and followed by the German Landrace pig which yielded pale skin animals. The Göttingen strain is a white non-pigmented small-sized miniature pig with an adult body weight of 30–40 kg [54]. All animals received humane care in compliance with the guide for the care and use of laboratory animals [55].

### Cells, cell culture conditions, and cell growth kinetics

Porcine fibroblast cells isolated from the ears of two 1-day-old female miniature pigs (MPF) and two 1-day-old female domestic pigs (PF) were grown and maintained in DMEM/high glucose (Hyclone) media enriched with 10% FBS (Hyclone), 1% penicillin-streptomycin (Gibco), and 2 mM L-glutamine (Gibco). To determine cellular lifespans in this study, primary MPF and PF cells were plated at a density of 3 × 10^5 ^cells/10 cm dish and passaged every 3 days following the standard 3T3 protocol; the number of population doublings per day was calculated.

Cell growth and growth rates were determined by plating cells at a density of 1 × 10^4 ^cells in 10% FBS and 2.5 × 10^4 ^cells in 0.5% FBS in 6-well plates and staining with a 0.01% crystal violet solution every other day for 6 days. Crystal violet was extracted from the stained cells using 10% acetic acid and subjected to spectrophotometric analysis (595 nm) to determine relative cell growth rates [56]. To assess responses to DNA damage, relatively early passage (P4) and late passage (P60) cells were treated with doxorubicin (0, 1, 3, and 5 uM)) for 24 h, and their viability measured by the standard trypan blue exclusion method.

### Senescence-associated β-galactosidase assay

To conduct the senescence-associated β-galactosidase activity assay, cells were fixed with 0.5% glutaraldehyde (pH 7.2) and washed in PBS (pH 7.2) supplemented with 1 mM MgCl_2 _and then stained in X-gal solution (1 mg/mL X-gal, 0.12 mM K_3_Fe [CN]_6_, 0.12 mM K_4_Fe [CN]_6_, 1 mM MgCl_2 _in PBS at pH 6.0) overnight at 37°C. After washing with phosphate-buffered saline, the plates were viewed by light microscopy.

### Plasmids, retroviral infection, and transfection

Cells were infected with replication-defective retroviruses produced by a PT67 amphotropic packaging cell line (Clontech) that had been transfected with pBabe-SV40LT-puro vector. Supernatants of the stable transfected PT67 cells (>70% confluent) were filtered through a 0.45 um filter to remove cellular debris and used to infect the cells three times at 12 h internals. The infected cells were selected with 2 ug/mL puromycin (Clontech) for 7 days. Cells were transfected with the pCI-hTert-neo vector and selected with 500 ug/mL neomycin (G418, Gibco) for 2 weeks.

### Western blotting

Cell extracts were prepared using a RIPA lysis buffer containing 1× protease inhibitor cocktail (Roche). Proteins in the cell extracts (50 ug) were separated on a 4~12% pre-cast SDS-PAGE gradient (Invitrogen) and transferred to PVDF membranes (Millipore). The membranes, which had been blocked with 5% skim milk, were incubated with anti-p53 antibody (sc-126, Santa Cruz Biotechnology, 1:500 dilution), anti-p21^WAF1 ^antibody (sc-397, Santa Cruz Biotechnology, 1:200 dilution), and anti-α-tubulin antibody (T9026, sigma, 1:5000 dilution), followed by incubation of HRP conjugated anti-mouse IgG and anti-rabbit IgG secondary antibody (Pierce). The immunoblot signals were detected with a SuperSignal West Pico kit (Pierce).

### Soft-agar assay

To measure cell anchorage independence, the primary MPF and PF cells (1 × 10^4^) were cultured in 6-well soft-agar dishes (1.6% and 0.7% bottom and top agar, respectively) for 3 weeks.

### Karyotyping

The mitotic chromosomes of MPF and PF cells were obtained following standard methods with slight modifications. Thus, cells were initially plated in 10% FBS containing DMEM for 48~72 h, treated with 0.01 mg/mL colcemid (Gibco), lysed for 15 min in a hypotonic solution, and fixed in a methanol:acetic acid (3:1) solution. Chromosomes were stained with 4% Giemsa solution in Gurr's buffer and the number of chromosomes in metaphase (n = 100 cells) from each cell line were determined.

### Statistical analysis

All experiments were replicated three times at least. Statistical significance was assessed by ANOVA, followed by Duncan's test in SAS software package (Ver.9.1).

## Authors' contributions

H-Y.O. and X.J. carried out isolation of the cells, cell growth analysis, western blotting, soft-agar assay, participated in the design and coordination of the study, and drafted the manuscript. J-G.K carried out RT-PCR analysis and conceived of the study. M-J.O., X.P., and Y.S.L carried out plasmid construction, transduction assay and telomerase activity analysis. J-M.K, M-S.Y. and C-I.S. participated pig growth study in vivo. K-C.H., H.K., Y-J.C. participated in the design and coordination of the study. K.Y.W. participated in the design and coordination of the study and helped draft the manuscript. All authors read and approved the final manuscript.
